# Transmission measurement at the Bernina branch of the Aramis Beamline of SwissFEL

**DOI:** 10.1107/S1600577519013237

**Published:** 2019-10-23

**Authors:** Pavle Juranic, Kai Tiedtke, Shigeki Owada, Takahiro Tanaka, Ulf Jastrow, Andrey Sorokin, Luc Patthey, Roman Mankowsky, Markus Degenhardt, Yunieski Arbelo, Christopher Arrell, John Smedley, Jen Bohon, Rolf Follath

**Affiliations:** a Paul Scherrer Institut, Villigen 5232, Switzerland; b DESY, Notkestrasse 85, 22607 Hamburg, Germany; c Japan Synchrotron Radiation Research Institute (JASRI), 1-1-1 Kouto, Sayo-cho, Sayo-gun, Hyogo 679-5198, Japan; d National Institute of Advanced Industrial Science and Technology (AIST), NMIJ, Tsukuba 305-8568, Japan; e Los Alamos National Laboratory, Los Alamos, NM 87545, USA

**Keywords:** instrumentation, performance, XFEL

## Abstract

The transmission of the optical components of the Bernina branch of the Aramis beamline at SwissFEL has been measured with an X-ray gas monitor from DESY and compared with a PSI gas detector upstream of the optical components. The transmission efficiencies of the Mo, Si and SiC mirror coatings of the Aramis beamline and the various other in-beam components were evaluated and compared with theoretical calculations, showing an agreement of 6% or better in all cases. The experiment has also shown the efficacy of the high-harmonic rejection mirrors at the Bernina branch of the Aramis beamline at SwissFEL, and characterized the transmission efficiency of the on-line spectrometer in the Aramis beamline. The theoretical transmission of the mirror coatings match the experimental data to within 7%. The accuracy of these measurements was checked against a radiative bolometer from a Japanese collaboration and found to agree to a level of 4% or better. Further comparisons with a diamond detector from a US-based inter-institute collaboration demonstrated a good agreement for the attenuator settings of the beamline.

## Abstract 2

The transmission of the optical components of the Bernina branch of the Aramis beamline at SwissFEL has been measured with an X-ray gas monitor from DESY and compared with a PSI gas detector upstream of the optical components. The transmission efficiencies of the mirrors and the various other in-beam components were evaluated and compared with theoretical calculations.

## 1. Introduction   

The Swiss Free Electron Laser (SwissFEL) (Milne *et al.*, 2017 ▸) is the newest hard X-ray FEL facility open to users. SwissFEL aims to provide short pulse lengths at high fluences to users for experiments in chemistry, biology, material sciences, spectroscopy and other fields. The optical layout of the beamline was created to deliver the photons as efficiently as possible, and with as few losses as possible, to the endstations. This paper presents the measurements of the transmission of the Bernina branch of the SwissFEL Aramis beamline. The study uses gas-based pulse energy measurements before the first mirror, with the photon beam intensity gas (PBIG) detector (Juranić *et al.*, 2018 ▸) and a mobile X-ray gas monitor (XGM) from DESY (Tiedtke *et al.*, 2008 ▸, 2014 ▸) placed at the end of the Bernina branch endstation, behind all of the optical components. The PBIG and the XGM detectors have the benefit of being near-identical copies, with the same working principle, dimensions, gas type and measurement devices. Both devices were constructed by the DESY photon diagnostics group, and then tested and calibrated at the Metrology Light Source of the Physikalisch-Technische Bundesanstalt (PTB) in Berlin (Gottwald *et al.*, 2012 ▸, 2019 ▸; Sorokin *et al.*, 2019 ▸) before installation of the PBIG at PSI. As a result of these preparations, the two devices were well correlated and calibrated against each other before their installation, ensuring that the beamline transmission measurements made were accurate. Additional cross-checks of the devices were made with a diamond detector from a collaborative group in the USA that includes the Los Alamos National Laboratory, Brookhaven National Laboratory, Argonne National Laboratory, Stony Brook University and SYDOR Technologies (Bohon *et al.*, 2010 ▸), and a room-temperature radiometer (Tanaka *et al.*, 2015 ▸, 2017 ▸) provided by the National Institute of Advanced Industrial Science and Technology (AIST) in Japan.

## 2. Setup   

**Figure 1:**
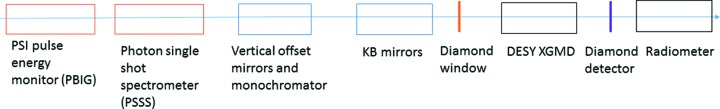
Layout of the experiment.

The layout of the experiment is shown in Fig. 1 ▸. The FEL photons travel from the undulators into the PBIG device, an integral part of the beamline and the main pulse energy diagnostic for SwissFEL. The device was filled with Xe gas that was photoionized by the passing X-rays, and the subsequently created ions were then extracted onto a split electrode that measured the currents with about an 11 s integration time constant through calibrated 6514 Keithley electrometers. The ionization chamber has a pusher voltage of about 4 kV and an extraction voltage on the ion side of about −150 V, while the gas pressure was kept at about 1 × 10^−4^ mbar. The current on the electrodes is used to calculate the number of photons per second and per pulse according to the equations in previous work (Tiedtke *et al.*, 2008 ▸, 2014 ▸; Sorokin *et al.*, 2019 ▸). This PBIG measurement was taken as the baseline for the beamline transmission calculations. The FEL was set to 6.08 keV photon energy and had a pulse energy between 100 µJ and 200 µJ.

The Aramis beamlines feature the photon single shot spectrometer (PSSS) (Rehanek *et al.*, 2017 ▸) which has the option of having a 100 nm grating inserted into the beam. The grating has a thickness of 10 µm with a 1 µm pitch and a fractional grating area of 0.5. The transmission through the grating was measured for consistency with theory, and the spectrometer was used to determine the photon energy for the transmission calculations. The spectrometer and its grating were removed from the beam to measure the efficiency of optical components further downstream.

**Figure 2:**
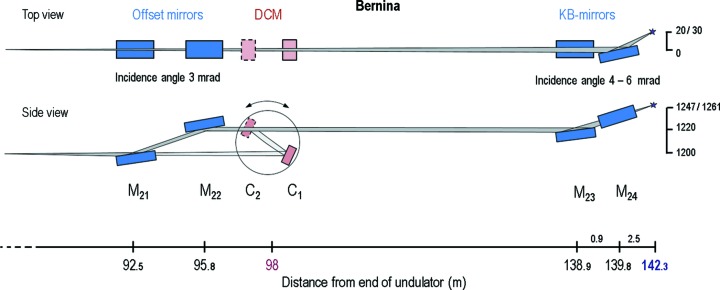
Optical layout of the Bernina branch of the Aramis beamline at SwissFEL.

Beyond the PSSS is the first optical element of the Aramis beamlines. The Bernina branch of the Aramis beamline is a hard X-ray beamline typically operating between photon energies of 4 keV and 12 keV. The optical layout is shown in Fig. 2 ▸ and described in more detail by Follath *et al.* (2016 ▸). The beamline consists of beam offset mirrors, a double-crystal monochromator and a pair of Kirkpatrick–Baez (KB) (Kirkpatrick & Baez, 1948 ▸) mirrors for focusing. The offset mirrors are always in the beam path, whereas the KB optics may or may not be inserted. The transmission measurements were made with both KB mirror configurations (inserted and not inserted into the beam).

**Figure 3:**
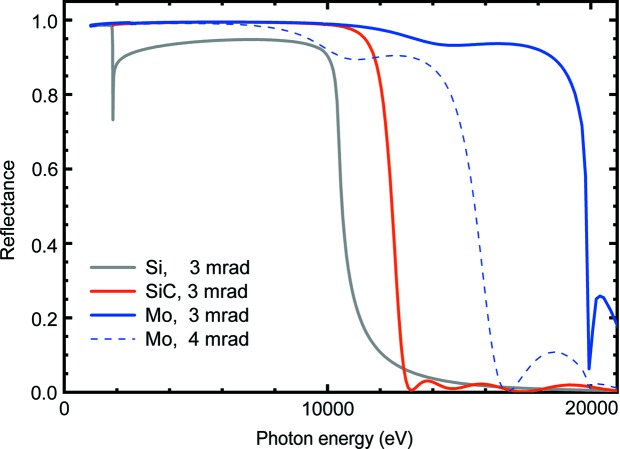
Reflectance of the three coatings Si, SiC and Mo on the offset mirrors at 3 mrad incidence angles and of coating Mo with a 4 mrad incidence angle on the KB mirrors.

The offset mirrors have three different coatings, pure silicon, 10 nm B_4_C on 36 nm SiC, and 15 nm B_4_C on 20 nm Mo; however, the KB mirrors only have a coating of Mo. The offset mirrors operate with a 3 mrad incidence angle, whereas the KB mirrors were used with an incidence angle of 4 mrad. The reflectance is calculated with optical constants from the Henke tables (Henke *et al.*, 1993 ▸) and displayed in Fig. 3 ▸ as a function of the photon energy. A surface roughness of 0.5 nm (r.m.s.) is assumed for the bulk material and all layers. The Si and SiC coatings of the offset mirrors can be used to suppress the higher harmonics above 6 keV and 7 keV, respectively. A 50 µm-thick diamond window (density 3.53 g cm^−3^) after the KB mirrors separates the beamline from the experimental stations. The values of mirror reflectance and diamond transmission for a fixed photon energy of 6.08 keV are listed in Table 1 ▸. The size of the beam at the exit of the KB mirrors is about 750 µm full width at half-maximum (FWHM), and is focused down to 2 µm at a distance of 3.6 m from the middle of the last KB mirror.

The DESY XGM was set up behind the diamond window at the end of the beamline, about 1.6 m behind the middle of the last KB mirror, making the beam size about 350 µm FWHM. Like the PBIG detector at the front of the whole beamline, it was filled with Xe gas to a pressure of about 6  × 10^−5^ mbar, its pusher mesh voltage was at 4000 V and its extraction plate voltage was at −168 V. The DESY XGM has an approximate 11 s time integration constant for its current measurements. A diamond detector (Bohon *et al.*, 2010 ▸) and a radiometer (Tanaka *et al.*, 2017 ▸, 2015 ▸) were installed for cross-calibration purposes downstream of the XGM.

The diamond detector was used to cross-calibrate transmission through the beamline attenuators, providing access to measurements below the sensitivity of the XGM for calibration of more absorbing filters. The diamond detector was fabricated using a 20 µm single-crystal electronic grade diamond substrate, patterned with nitrogen-incorporated ultra-nanocrystalline contacts as described previously (Zou *et al.*, 2018 ▸), and presented a 3 mm active area for beam measurement. The beam size at the detector was estimated to be about 200 µm FWHM.

The radiometer, which is designed for absolute power measurements of the FEL, is equipped with an absorber, the absorptance of which is more than 0.997 in the photon energy range from 10 eV to 60 keV (Tanaka *et al.*, 2015 ▸, 2017 ▸). The absorber is a cavity type that consists of a tungsten plate and copper cylinder, and was kept at a constant temperature with an electrical heater. The absolute laser power of an FEL beam is measured by the electrical power difference measurement with and without the FEL beam. The time constant of the radiometer is approximately 10 s, and therefore the radiometer provides the average laser power of the FEL. Both the radiometer and the diamond detector were used to confirm the reliability and accuracy of the gas-based detection method used by the PBIG and the XGM with measurement methods based on different principles and properties. The beam size at the radiometer was estimated to be about 150 µm FWHM.

## 3. Results   

We measured the transmission of the beamline in pink mode at a photon energy of a 6.08 keV and a 100–200 µJ pulse energy. The photon energy was determined beforehand with the monochromator and the PSSS, which were in agreement to 10 eV or better. The beamline was then switched to pink beam mode and the pulse energy was measured at the end of the beamline for all combinations of the three coatings of the two offset mirrors and with the KB mirrors inserted, as shown in Fig. 3 ▸. The PSSS was retracted from the beam for the measurements meant to characterize only the transmission of the mirrors. The diamond detector was removed from the beam for the PBIG/XGM/radiometer comparison measurements, and was separately used in tandem with the XGM and with the always-on PBIG for the calibration of the attenuators.

The beamline transmission at 6.08 keV for Si coatings on the offset mirrors with the KB removed was 0.77 ± 0.03 (calculated: 0.748). The measured and expected transmission values of the mirror combinations are given in Table 2 ▸. The Si coatings seem to match the theoretical values well and are typically within 2.5% of the expected value, whereas the performances of the SiC and Mo coatings were slightly worse, being up to 6% below the theoretical transmission value. The coatings are used to suppress second-order light at different photon energies, as shown in Fig. 3 ▸, and the most often used coating at low photon energies is Si. The difference in the accuracy between symmetric measurements such as the SiC/Mo and Mo/SiC combinations comes from the variation of the pulse energy for the repeated measurements. For example, the Mo/SiC pulse energy measurements had a standard deviation of 3.6% over the measurement period, whereas the SiC/Mo measurements had a more stable beam with only a 2.3% standard deviation.

We also performed a comparison of average pulse energies between the XGM and radiometer at 6 keV and 7 keV. Though initial values from the radiometer showed about 10% more flux than the XGM, an alignment issue with the radiometer was discovered. The radiometer, when misaligned, could cause the FEL beam to hit the Cu cylinder which the temperature sensor is attached to, giving an inaccurate reading of the beam power. After the issue was discovered, and the alignment corrected and re-measured, the values between the XGM and the radiometer matched very well, though the radiometer was not used for the transmission measurements. As shown in Table 3 ▸, the comparison results show agreement within 5% of each other without any dependence on photon energy, repetition rate and the target gas in the XGM. This independent evaluation of the XGM accuracy confirms that the transmission measurements are of good quality.

The transmission efficiency of the PSSS grating was also characterized. The PSSS uses a diamond grating with a 50/50 gap/hole ratio, a depth of about 1 µm and a supporting layer of 10 µm. The expected measurement of the transmission of the zeroth-order at 6.08 keV is 0.907 according to calculations performed by the Center for X-ray Optics software (http://henke.lbl.gov/optical_constants/tgrat2.html); the measured transmission was 0.91 ± 0.02. This discrepancy falls well within the tolerances of the grating manufacturing process.

The solid attenuators in the beamline were characterized with the diamond detector and the DESY XGM measuring the flux at the end of the beamline *versus* the flux measured by the PBIG detector in the front-end at 6.08 keV photon energy. The diamond detector was biased at +40 V for the measurements. The device is capable of sub-nanosecond response, but was used in averaging mode for the cross-calibration.The agreement between the diamond and the XGM was better than 3% for attenuators with transmission >1%, whereas the accuracy of the devices dropped and deviated from each other for lower transmissions. Both devices deviate from the CXRO calculations. This is probably due to actual deviations in the attenuator thicknesses. A summary of a few of these measurements is shown in Table 3 ▸. The discrepancy at lower transmission values is likely to be due to the high relative content of higher harmonics with respect to the main 6 keV beam, and the difference in response of the two detectors to it. Though the offset mirror suppresses the higher harmonics, an attenuator overwhelmingly favors attenuating the fundamental photon energy with respect to the higher harmonics. An attenuator allowing 5% transmission at 6 keV still typically allows 95% or more transmission of the third harmonic of 18 keV, raising the relative harmonic content to a level that is comparable with the fundamental and compensating for the offset mirror suppression. The transmission comparisons between the XGM and the diamond detectors were carried out with pulse energies between 110 µJ and 200 µJ (Table 4 ▸).

## 4. Conclusions   

The cross-calibration measurements with the DESY-provided XGM detector and the installed PBIG device have been successfully performed at the Bernina branch of the Aramis beamline of SwissFEL. The experiment measured the transmission of the offset mirrors, spectrometer components and KB mirrors at a photon energy of 6.08 keV. The experimental data are in good agreement with theoretical values. The built optical components of the Bernina branch of the Aramis beamline are performing to the specifications set out, and appear to be delivering light with the expected efficiency and transmission.

## Figures and Tables

**Table 1:** Reflectance (*R*) of the coatings and transmission (*T*) of the diamond window at the end of the beamline for 6.08 keV

	Coating				
	Si	SiC	Mo	Mo (4 mrad)	50 µm diamond
*R*	0.9463	0.9944	0.9949	0.9896	–
*T*	–	–	–	–	0.8348

**Table 2:** Transmission through the beamline for all combinations of the offset mirror coatings and with the KB mirrors inserted Measured and calculated (in brackets) values for a photon energy of 6.08 keV. At this photon energy the diamond window dominates the overall losses.

		Coating on M_22_		
		Si	SiC	Mo
Coating on M_21_	Si	0.73 ± 0.03	0.76 ± 0.03	0.76 ± 0.04
		(0.732)	(0.769)	(0.770)
	SiC	0.73 ± 0.03	0.76 ± 0.02	0.76 ± 0.04
		(0.769)	(0.808)	(0.809)
	Mo	0.75 ± 0.03	0.78 ± 0.02	0.77 ± 0.03
		(0.770)	(0.809)	(0.809)

**Table 3:** Comparison of average pulse energies between the XGM and the radiometer

		Average pulse energy (µJ)		
Photon energy (keV)	Repetition (Hz)	XGM (target gas)	Radiometer	Ratio
6.08	10	124.0 (Xe) ± 6.0	124.5 ± 6.9	1.015
6.08	25	152.1 (Xe) ± 4.0	149.9 ± 7.1	0.996
7.22	25	126.0 (Xe) ± 3.3	130.4 ± 6.1	1.010
7.27	25	122.8 (Kr) ± 4.8	121.5 ± 6.1	0.967

**Table 4:** Transmission comparison between the XGM and diamond detectors

Attenuator (material and thickness in µm)	Transmission at XGM	Transmission at diamond detector	Theoretical transmission (CXRO)	XGM/diamond detector deviation
Si30	0.2944	0.3010	0.3860	0.022
C100 + Si100	0.0232	0.0237	0.0290	0.025
Si100 + Si20 + Si30	0.0044	0.0055	0.0085	0.244
